# Calcium Chloride in Neonatal Parenteral Nutrition Solutions with and without Added Cysteine: Compatibility Studies Using Laser and Micro-Flow Imaging Methodology

**DOI:** 10.1371/journal.pone.0136894

**Published:** 2015-08-28

**Authors:** Robert K. Huston, J. Mark Christensen, Sultan M. Alshahrani, Sumeia M. Mohamed, Sara M. Clark, Jeffrey A. Nason, Ying Xing Wu

**Affiliations:** 1 Northwest Newborn Specialists, PC and Pediatrix Medical Group, Portland, OR, United States of America; 2 Department of Pharmaceutical Sciences, College of Pharmacy, Oregon State University, Corvallis, OR, United States of America; 3 Neonatal Pharmacy, Providence St. Vincent Medical Center, Portland, OR, United States of America; 4 School of Chemical, Biological, & Environmental Engineering, Oregon State University, Corvallis, OR, United States of America; 5 Medical Data Research Center, Providence Health and Services, Portland, OR, United States of America; University of Florida, UNITED STATES

## Abstract

**Background:**

Previous studies of compatibility of calcium chloride (CaCl_2)_ and phosphates have not included particle counts in the range specified by the United States Pharmacopeia. Micro-flow imaging techniques have been shown to be comparable to light obscuration when determining particle count and size in pharmaceutical solutions.

**Objective:**

The purpose of this study was to do compatibility testing for parenteral nutrition (PN) solutions containing CaCl_2_ using dynamic light scattering and micro-flow imaging techniques.

**Methods:**

Solutions containing TrophAmine (Braun Medical Inc, Irvine, CA), CaCl_2_, and sodium phosphate (NaPhos) were compounded with and without cysteine. All solutions contained standard additives to neonatal PN solutions including dextrose, trace metals, and electrolytes. Control solutions contained no calcium or phosphate. Solutions were analyzed for particle size and particle count. Means of Z-average particle size and particle counts of controls were determined. Study solutions were compared to controls and United States Pharmacopeia (USP) Chapter 788 guidelines. The maximum amount of Phos that was compatible in solutions that contained at least 10 mmol/L of Ca in 2.5% amino acids (AA) was determined. Compatibility of these solutions was verified by performing analyses of 5 repeats of these solutions. Microscopic analyses of the repeats were also performed.

**Results:**

Amounts of CaCl_2_ and NaPhos that were compatible in solutions containing 1.5%, 2%, 2.5%, and 3% AA were determined. The maximum amount of NaPhos that could be added to TrophAmine solutions of > = 2.5% AA containing at least 10 mmol/L of CaCl_2_ was 7.5 mmol/L. Adding 50 mg/dL of cysteine increased the amount of NaPhos that could be added to solutions containing 10 mmol/L of CaCl_2_ to 10 mmol/L.

**Conclusion:**

Calcium chloride can be added to neonatal PN solutions containing NaPhos in concentrations that can potentially provide an intravenous intake of adequate amounts of calcium and phosphorus.

## Introduction

In 1997 Bishop et al reported results of a randomized controlled trial comparing outcomes for infants who received parenteral nutrition (PN) solutions containing calcium gluconate (CaGlu) versus calcium chloride (CaCl_2_) that found impaired neurological development of neonates due to increased aluminum (Al) exposure from solutions containing CaGlu [1]. A follow up study associated the high Al exposure from PN solutions containing CaGlu with reduced bone mass in adolescence [2]. A recent study suggests that Al exposure from CaGlu containing PN solutions also presents a risk to adults receiving long-term PN with regard to bone mineralization [3]. The American Society for Parenteral and Enteral Nutrition (A.S.P.E.N.) acknowledges that Al contamination of PN solutions is a risk factor for metabolic bone disease of preterm infants and recommends that “efforts be made to reduce the aluminum content of PN” [4].

Aluminum is a contaminant introduced into many small and large volume parenteral products during the manufacturing process, as well as due to leaching of Al during sterilization of glass containers [5]. Currently, the FDA recommends limiting the Al intake, inadvertently present due to contamination of PN solutions, for preterm infants to ≤0.19 μmol/kg/day (≤5 μg/kg/day) [6]. Poole et al have shown that it is not possible to meet this goal and provide a parenteral intake of adequate amounts of calcium (Ca) and phosphorus using neonatal PN solutions containing CaGlu [7]. Calcium chloride is the only calcium additive available in North America that, when added to neonatal PN solutions, limits inadvertent administration of Al to levels near the FDA recommendation [1, 8]. Calcium gluconate in plastic vials, as opposed to glass vials, has been available in Europe, but not North America, and is as low in Al content as CaCl_2_ [9].

Calcium gluconate has been the preferred Ca additive in North America for PN solutions due to decreased dissociation of calcium cations into solution, thereby decreasing the risk of forming precipitates with phosphate, compared to CaCl_2_ [10]. A recent study of Ca and phosphate (Phos) compatibility in neonatal PN solutions utilized laser light dynamic light scattering (DLS) methodology to evaluate compatibility of CaCl_2_ and potassium phosphate (KPhos) [11]. Guidelines outlined by the United States Pharmacopeia (USP) chapter 788 specify acceptable particle counts for particles > = 10 microns and > = 25 microns in large volume parenteral solutions [12]. These guidelines recommend using laser light obscuration (LO) methodology or light microscopic techniques to detect particles > = 10 microns. For LO, suggested limits for particles > = 10 microns are 25 particles per mL and for particles > = 25 microns are 3 particles per mL. For microscopic analysis, suggested limits for particles > = 10 microns are 12 particles per mL and for particles > = 25 microns are 2 particles per mL. Studies demonstrate that DLS is very accurate when evaluating particle sizes <1300 nm (1.3 microns) compared to LO whereas LO is more accurate for particles of larger diameters [13]. Micro-Flow imaging (MFI) techniques have more recently been shown to be comparable to light obscuration when determining particle count and size for spherical, opaque particles >2 microns but more accurate when analyzing particles that are non-spherical or having a refractive index similar to the solvent solution [14–15].

A previous study, performed in 2013 [11], attempted to evaluate compatibility of CaCl_2_ and sodium phosphate (NaPhos) in solutions of TrophAmine, however, not all concentrations of amino acids (AA), Ca, and Phos could be evaluated due to a national shortage of NaPhos. The purpose of the study described in this manuscript was to continue the compatibility testing of neonatal PN solutions containing CaCl_2_ and NaPhos, including repeating solutions studied in 2013, and to include the evaluation of solutions containing cysteine compared to solutions without cysteine. In addition to visual and DLS scattering methods that were used previously, micro-flow imaging methodology as well as the USP recommended microscopic techniques for particle counts were included to verify compatibility.

## Methods

Two studies were conducted from February through October, 2014. All study solutions were compounded by a neonatal pharmacist in the Neonatal Intensive Care Unit (NICU) pharmacy at Providence St. Vincent Medical Center in clear plastic Exacta Mix 250 mL EVA containers (Baxa Corporation, Englewood, CO) using a Baxa Exactamix 2400 Compounder (Baxa Corporation, Englewood, CO). Additives that were included in all solutions are shown in Table 1. The final volume of each solution was 100–200 mL.

**Table 1:** Standard Parenteral Nutrition Additives for All Solutions Compounded in the Precipitation Studies.

Additive	Manufacturer	Total Dose (per dL)
Dextrose 70%	Hospira, Inc., Lake Forest, IL	10 g
Sterile Water	Hospira, Inc., Lake Forest, IL	QS to 100 mL
Heparin (1000 units/mL)	APP Pharmaceuticals, Schaumburg, IL	50 units
Magnesium Sulfate 50% (4 mEq/mL)	APP Pharmaceuticals, Schaumburg, IL	0.5 mEq
Zinc Chloride (1 mg/mL)	Hospira, Inc., Lake Forest, IL	400 μg
Copper Sulfate (400 mcg/mL)	American Regent, Inc., Shirley, NY	20 μg
Selenium (40 μg/mL)	American Regent, Inc., Shirley, NY	2 μg
Levocarnitine (200 mg/mL)	Sigma-Tau Pharmaceuticals,Gaithersburg, MD	5 mg
Sodium Acetate (2 mEq/mL)	Hospira, Inc., Lake Forest, IL	2 mEq
Potassium Chloride (2 mEq/mL)	Hospira, Inc., Lake Forest, IL	1 mEq

In study 1 solutions containing TrophAmine (Braun Medical Inc., Irvine, CA) in concentrations of 1.5%, 2%, 2.5%, and 3% AA were compounded with and without cysteine (Sandoz Inc, Princeton, NJ) in a concentration of 50 mg/dL. Calcium chloride (Hospira Inc, Lake Forest, IL or International Medication Systems, South El Monte, CA) was added in concentrations of 2.5, 5, 7.5, 10, 12.5, and 15 mmol/L while NaPhos (APP Pharmaceuticals, Shaumberg, IL or Lloyd Center Compounding Pharmacy, Portland, OR) was added in concentrations of 5, 7.5, 10, 12.5, and 15 mmol/L. Concentrations were studied in order from lowest to highest concentration. Based upon results for lower AA concentrations not all concentrations of CaCl_2_ and NaPhos were studied at each AA concentration. For example, if solutions containing specific concentrations of Ca and Phos were compatible in AA concentrations of 1.5% and 2% AA, additional compatibility testing for these concentrations of Ca and Phos in 2.5% and 3% AA was not performed. Likewise, not all compatible combinations in PN solutions without cysteine were studied when adding cysteine. Finally, based upon data from previous experience [8, 11], if it was unlikely that certain concentrations of Ca and Phos would be compatible in low AA concentrations those combinations of additives were not studied. For each AA concentration at least two controls without any added CaCl_2_ or NaPhos were compounded. At least two solutions were also compounded for each AA concentration with and without cysteine that contained 12.5 mmol/L of CaCl_2_ and 20 mmol/L of NaPhos (concentrations that previous experience found by visual examination to clearly precipitate).

After compounding, solutions were transported in the PN bag by automobile (time for transport: 1.5 hr) to the Department of Pharmaceutical Sciences at Oregon State University where they were incubated at 37°C for 24 hours in a warming oven. Bags were visualized in a dark room while trans-illuminating the solution with a bright beam of light before and after incubation to determine evidence of precipitation. Solution pH was measured before and after incubation using a pH meter (Mettler Toledo FIVE, Schwerzenbach, Switzerland). After incubation, three samples from each bag were analyzed using a laser instrument (Zetasizer Nano ZS, Model ZEN3600, Malvern Instruments Ltd, Worcestershire, UK) to determine the Z-average particle size of the solutions as described previously [11]. In addition, samples of each solution were analyzed for particle counts using a micro-flow imaging instrument (Model DPA 4100, Brightwell Technologies, Inc., Ottawa, Ontario, Canada). Solutions that were determined to have acceptable concentrations of Ca and Phos were prepared and analyzed again at least once, or confirmed by results from at least one more solution that had higher concentrations of Ca and/or Phos in the same AA concentration or similar concentrations of Ca and Phos in a solution with a lower AA concentration to verify compatibility.

Data for control solutions was entered into an Excel spreadsheet (Microsoft Corp., Redmond, WA) and means, standard deviations (SD), medians, and range for each measurement were determined using the Excel Spreadsheet software. Incompatible solutions were defined as those with visual evidence of precipitation, those with average particle counts for particles > = 10 microns or > = 25 microns that exceeded the mean plus 2 standard deviations of the control solutions, or those with a Z-average particle diameter>1000 nm and also average particle counts that exceeded United States Pharmacopeia (USP) Chapter 788 guidelines for LO [12]. For study solutions that were replicated, all values were averaged for comparison to control values. Solutions from 2013 and the current study were included when evaluating compatibility in Study 1.

Study 2 was conducted in order to verify the maximum concentration of NaPhos that can be added to solutions containing 2.5% AA and 10 mmol/L of CaCl_2_. These concentrations of AA and Ca were chosen because a concentration of 2.5% AA or more is most often used clinically in the NICU and a Ca concentration of 10 mmol/L is a minimum amount that can provide close to the recommended Ca intake at fluid volumes administered in the NICU. In this study replicate PN solutions were compounded containing the maximum amounts of NaPhos that appeared compatible, as defined above, in Study 1 for solutions containing 2.5% AA, with and without added cysteine, and a CaCl_2_ concentration of 10 mmol/L. Five solutions each with no added Ca or Phos, with 10 mmol/L of Ca only, with the two concentrations of Phos only, and with each concentration of Phos plus Ca of 10 mmol/L, with and without cysteine were compounded. Thus, there were 12 study solutions, repeated 5 times, for a total of 60 solutions compounded for this portion of the study. In this study, in addition to the Z-average particle size determined by the Zetasizer and particle counts determined by MFI, microscopic particle counts were determined using procedures recommended by the USP Chapter 788 [12]. Microscopic counts were verified by two investigators.

Statistical analysis was performed by a statistician using PASW 17 (SPSS Inc., Chicago, IL) and R 3.0 (http://www.R-project.org) software. Means and standard deviations of the 5 replicated solutions for each of the 12 combinations of additives were computed and compared. Analysis of variance (ANOVA) was used to verify significant trends. Analyses that were compared were: Z-average particle size as determined by the Zetasizer; MFI particle counts > = 5 microns, > = 10 microns, and > = 25 microns; and microscopic particle counts > = 2 microns, > = 10 microns, and > = 25 microns.

The studies were approved by the Institutional Review Board for Providence Health and Services, Portland, Oregon. No formal review was required since no human subjects were involved.

## Results

### Study 1

For 29 controls (containing no Ca or Phos), the mean ± SD of pH prior to incubation was 5.92±0.19 and after incubation was 5.95±0.14. Mean and standard deviation (median: range) of the Z-average particle diameter (N = 28 controls) was 417+/-267 (368: 39–1072 nm). For 25 control solutions, MFI particle counts > = 10 microns were 30+/-26 (23: 0–85 particles/mL), and > = 25 microns were 1.6+/-2.2 (1: 0–7 particles/mL). The results of the measured pH of the study solutions for Study 1 are shown in Table 2. For solutions without cysteine, there were a total of 138 study solutions of which 46 were compounded in 2013 and 92 were compounded in 2014. Of the 92 in 2014, there were 32 compounded solutions that were replicates. Of the 46 PN solutions compounded in 2013, 26 were replicated in 2014. For study solutions containing cysteine, all 88 were compounded in 2014 and there were 20 solutions that were replicates. The maximum concentrations of elemental Ca, as CaCl_2_, that were compatible with various concentrations of NaPhos and AA in solutions without and with cysteine added are shown in Tables 3 and 4.

**Table 2:** pH for Study Solutions in Study 1.

Year	% Amino Acids	N	pH (0 hr)	pH (24 hr)
2013 (No Cys)	1.5%	11	5.86±0.07	5.78±0.09
2013 (No Cys)	2%	11	5.82±0.04	5.78±0.05
2013 (No Cys)	2.5%	12	5.80±0.05	5.81±0.04
2013 (No Cys)	3%	12	5.75±0.04	5.81±0.04
2014 (No Cys)	1.5%	18	6.19±0.17	6.15±0.15
2014 (No Cys)	2%	20	6.15±0.17	6.09±0.15
2014 (No Cys)	2.5%	22	6.12±0.15	6.08±0.15
2014 (No Cys)	3%	32	6.09±0.18	6.07±0.16
2014 (Cys)	1.5%	18	5.87±0.06	5.81±0.07
2014 (Cys)	2%	18	5.89±0.11	5.82±0.06
2014 (Cys)	2.5%	26	5.79±0.14	5.85±0.10
2014 (Cys)	3%	26	5.86±0.10	5.80±0.08

Cys, cysteine.

**Table 3:** Maximum Concentrations (mmol/L)^a^ of Elemental Calcium (as CaCl_2_) Allowable in TrophAmine Solutions Containing Sodium Phosphate Without Cysteine.

Amino Acid	NaPhos	NaPhos	NaPhos	NaPhos	NaPhos
g/L (%)	5 mmol/L	7.5 mmol/L	10 mmol/L	12.5 mmol/L	15 mmol/L
15 (1.5%)	10	2.5	2.5	0	0
20 (2%)	10	5	5	2.5	0
25 (2.5%)	12.5	10	5	2.5	0
30 (3%)	12.5	10	5	2.5	2.5

CaCl_2_: calcium chloride; NaPhos: sodium phosphate.

^a^One mmol of calcium equals 40 mg or 2 mEq.

**Table 4:** Maximum Concentrations (mmol/L)^a^ of Elemental Calcium (as CaCl_2_) Allowable in TrophAmine Solutions Containing Sodium Phosphate With Added Cysteine (50 mg/dL).

Amino Acid	NaPhos	NaPhos	NaPhos	NaPhos	NaPhos
g/L (%)	5 mmol/L	7.5 mmol/L	10 mmol/L	12.5 mmol/L	15 mmol/L
15 (1.5%)	10	2.5	2.5	0	0
20 (2%)	10	5	5	2.5	0
25 (2.5%)	12.5	10	10	2.5	0
30 (3%)	12.5	10	10	2.5	2.5

CaCl_2_, calcium chloride; NaPhos, sodium phosphate.

^a^One mmol of calcium equals 40 mg or 2 mEq.

An additional 28 PN solutions were compounded containing 12.5 mmol/L of CaCl_2_ and 20 mmol/L of NaPhos. The mean and standard deviation (median: range) of the Z-average particle diameter (N = 15) was 6035+/-6975 (3766: 11–23567 nm). For 15 solutions, MFI particle counts per mL > = 10 microns were 10939+/-19331 (2665: 48–59431), and > = 25 microns were 1026+/-1958 (76: 0–5335). All but one of these solutions had evidence of visual precipitation. The one solution that did not precipitate had a Z-average particle size of 538 nm, MFI particle count > = 10 microns of 71 per mL, and MFI particle count > = 25 microns of 0. There were 5 solutions compounded for each AA concentration without cysteine and 2 for each AA concentration with cysteine. None of the solutions that had visual evidence of precipitation in which particle size data from both Z-average particle size and MFI measurements were obtained met criteria for compatibility, as described above, based upon the Z-average particle size and MFI particle counts.

### Study 2

In Study 1 the maximum compatible amount of NaPhos that could be added to solutions containing 2.5% AA and 10 mmol/L of CaCl_2_ for solutions without cysteine was 7.5 mmol/L and for solutions with cysteine was 10 mmol/L. Tables 5 and 6 present the results from Study 2 verifying the compatibility of PN solutions containing these nutrient concentrations. The only solution in Study 2 that did not meet our criteria for compatibility (as described in the Methods) was one of five solutions that contained 10 mmol/L of CaCl_2_ and 10 mmol/L of NaPhos without cysteine. This solution was cloudy on visual inspection after incubation and was the only solution that did not meet USP Chapter 788 criteria for particle counts for LO when analyzed using MFI. There was one solution that contained cysteine without Ca or Phos that appeared cloudy on visual inspection prior to incubation for unknown reasons but met criteria for compatibility based upon USP Chapter 788 guidelines for microscopic and LO particle counts. The Z-average particle size was also less than 1000 nm. There were two solutions that did not meet USP Chapter 788 criteria for compatibility based upon microscopic particle counts > = 25 microns of 3 per mL. Both of these solutions contained cysteine and NaPhos but only one contained CaCl_2_. Both solutions met USP Chapter 788 criteria for LO particle counts and had Z-average particle size <1000 nm.

**Table 5:** Compatibility Studies for 2.5% TrophAmine Solutions^a^ Containing CaCl_2_ and Sodium Phosphate Without Added Cysteine (Mean±SD).

Ca^b^	Phos	pH	pH	MFI^c^	MFI^c^	MFI^c^	Z-Ave	Micro^c^	Micro^C^	Micro^C^
mmol/L	mmol/L	0 hr	24 hr	≤5 μm	≥10 μm	≥25 μm	nm	≥2 μm	≥10 μm	≥25 μm
0	0	5.67±0.12	5.56±0.12	670±1052	7.4±7.6	0.0±0.0	298±157	24.0±23.9	2.8±2.0	0.6±0.5
0	7.5	5.81±0.06	5.68±0.04	654±1327	4.4±5.0	0.2±0.4	485±273	11.2±10.8	2.2±2.3	0.0±0.0
0	10	5.83±0.07	5.70±0.03	709±1305	4.8±7.0	0.4±0.89	406±418	11.8±11.4	3.8±04.8	1.2±1.3
10	0	5.66±0.06	5.57±0.07	352±635	1.6±2.3	0.0±0.0	455±357	16.8±13.9	2.2±2.2	0.4±0.5
10	7.5	5.78±0.07	5.72±0.09	1172±2301	10.2±7.9	0.0±0.0	880±883	11.8±5.2	2.0±1.4	0.4±0.5
10	10	5.80±0.07	5.68±0.05	1222±2262	10.8±12.0	0.4±0.5	711±652	14.8±8.0	1.8±1.6	0.4±0.9

Ca, calcium; CaCl_2_, calcium chloride; MFI, micro-flow imaging; Micro, microscopic; Phos, phosphate; SD, standard deviation; Z-Ave, Zetasizer Z-average particle diameter.

^a^N = 5 for all groups.

^b^One mmol of elemental calcium equals 40 mg or 2 mEq.

^c^Particle count per mL.

**Table 6:** Compatibility Studies for 2.5% TrophAmine Solutions^a^ Containing CaCl_2_ and Sodium Phosphate With Added Cysteine of 50 mg/dL (Mean±SD).

Ca^b^	Phos	pH	pH	MFI^c^	MFI^c^	MFI^c^	Z-Ave	Micro^c^	Micro^c^	Micro^c^
mmol/L	mmol/L	0 hr	24 hr	≥5 μm	≥10 μm	≥25 μm	nm	≥2 μm	≥10 μm	≥25 μm
0	0	5.48±0.09	5.36±0.08	501±804	5.0±8.0	0.0±0.0	362±158	12.6±7.6	1.2±1.1	0.4±0.9
0	7.5	5.58±0.11	5.46±0.11	288±574	1.8±2.39	0.0±0.0	426±190	10.0±4.8	0.8±1.3	0.4±0.5
0	10	5.62±0.09	5.48±0.10	485±982	3.8±5.3	0.2±0.4	320±234	13.8±7.5	1.6±0.9	0.6±0.5
10	0	5.44±0.09	5.31±0.07	507±1059	1.8±1.3	0.2±0.4	651±425	13.0±3.4	2.2±0.8	0.6±0.5
10	7.5	5.59±0.16	5.41±0.05	915±1169	3.2±3.1	0.0±0.0	619±254	18.0±12.9	3.0±2.7	1.2±1.3
10	10	5.57±0.10	5.24±0.47	986±1316	2.6±2.1	0.2±0.4	609±425	15.6±13.6	1.0±1.0	0.0±0.0

Ca, calcium; CaCl_2_, calcium chloride; MFI, micro-flow imaging; Micro, microscopic; Phos, phosphate; SD, standard deviation; Z-Ave, Zetasizer Z-average particle diameter.

^a^N = 5 for all groups.

^b^One mmol of elemental calcium equals 40 mg or 2 mEq.

^c^Particle count per mL.

**Fig 1:**
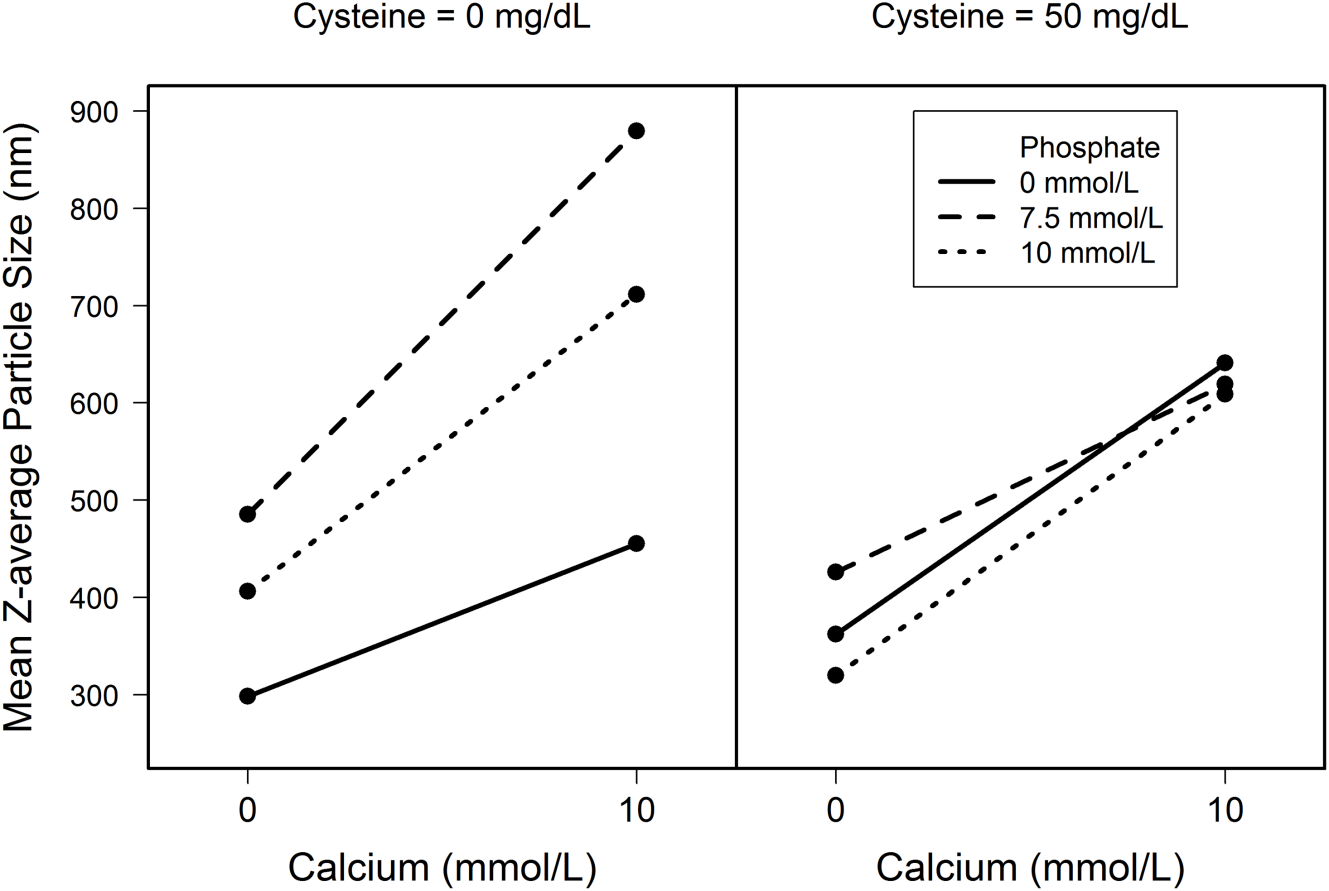
Mean Z-average particle size (nm) for solutions in Study 2. One mmol of elemental calcium equals 40 mg or 2 mEq.

Statistical analysis found no significant differences among groups of solutions based upon MFI or microscopic particle counts. There was a high degree of variability among the five samples for each group of solutions for MFI particle counts > = 5 microns but not for the other measurements. There was an increase in the Z-average particle size with the addition of calcium to PN solutions but no difference between solutions containing Ca only and those containing both Ca and Phos (Fig 1). The increase in the Z-average particle size associated with calcium was confirmed by ANOVA which found a significant positive association of Ca with Z-average particle size but no significant associations with Phos or cysteine (Table 7).

**Table 7:** Analysis of Variance for Z-average Particle Size.

Additive	p-Value
Calcium	0.012
Cysteine	0.679
Phosphate	0.442

## Discussion

This is the first study to analyze the compatibility of CaCl_2_ with NaPhos in neonatal PN solutions with and without added cysteine. As has been shown for solutions containing CaGluc, [16], more phosphate can be added to compounded PN solutions containing a given amount of CaCl_2_ when the solution also contains cysteine. This may be due to the decrease in pH of solutions containing cysteine compared to those without cysteine although it is possible that there may be some complex formation of Ca with cysteine as well. The pH effect is best demonstrated in Tables 5 and 6 from Study 2. In this study all solutions with and without cysteine were compounded on the same day, and the same lot of TrophAmine, having the same starting pH and AA configuration, was used for all solutions. Study 2 confirmed that the maximum amount of NaPhos that was compatible in solutions containing 2.5% AA and 10 mmol/L of elemental Ca as CaCl_2_ without added cysteine was 7.5 mmol/L. This is the same amount that was found to be compatible in an earlier study of compatibility of CaCl_2_ and KPhos [11]. Adding cysteine in a concentration of 50 mg/dL increased the amount of NaPhos that could be added to solutions containing 2.5% AA and 10 mmol/L of elemental Ca as CaCl_2_ to 10 mmol/L.

The need for more data on the compatibility of CaCl_2_ with Phos in PN solutions has been discussed previously [17–18]. The study reported here presents compatibility studies for neonatal PN solutions containing TrophAmine and CaCl_2_ using multiple methods including analysis of microscopic particle counts performed according to procedures recommended by the USP Chapter 788. The procedure preferred by the USP is LO [12]. MFI has become the preferred method for determining particle counts and morphology of protein particles and silicon oil droplets due to its improved accuracy over LO for determining the size and shape particles that may have a refractive index similar to the solvent. Latex spheres have a refractive index greater than water and similar to dibasic calcium phosphate (1.59, 1.33, and 1.59, respectively). Dibasic calcium phosphate is the precipitate of interest when performing compatibility studies for Ca and Phos in PN solutions. Comparison studies using spherical and elongated latex spheres demonstrate that MFI is as accurate as LO in performing particle counts of spheres but may be more accurate than LO for measuring particle size of non-spherical latex particles > = 10 microns in size [14,15,19,20]. Therefore, the published studies suggest that MFI should be just as accurate as LO when performing particle counts to detect calcium phosphate precipitates in PN solutions. Indeed, calibration procedures for both LO and MFI require validation measurements using latex particle standards.

The only other studies that have included evaluating particle counts compared to USP guidelines for PN solutions containing TrophAmine are two studies from the same center that evaluated one concentration of CaGluc and NaPhos in various AA concentrations [21–22]. Laser light obscuration methods were used to obtain particle counts in these studies. Particle counts for controls and compatible solutions in both Study 1 and Study 2 are within the ranges of those reported for compatible solutions in the studies that used LO to verify compatibility. Evaluation by light microscopy according to USP Chapter 788 guidelines [12] was also included in Study 2 to verify the maximum amounts of CaCl_2_ and NaPhos that were compatible in solutions containing 2.5% AA. In a recent study light microscopy appeared to be more sensitive when evaluating compatibility than LO or visual evidence of precipitation [23].

There are limitations to this study. Solutions that were compounded in 2013 were included in which MFI particle counts were not performed. Almost all of these solutions precipitated visually, however, and many of these solutions, including all that did not precipitate visually in 2013, were repeated in 2014. A single dextrose concentration of 10% was used for all solutions. When AA concentration has been controlled in previous studies no significant effect of dextrose concentration on Ca and Phos compatibility in PN solutions has been documented when comparing 10% versus 25% dextrose [24] or when comparing 5% versus 10% dextrose [21–22]. A concentration of 50 mg/dL of cysteine was used for all solutions tested containing cysteine. This is lower than has been used in studies of CaGluc. This dose was selected in order to minimize Al exposure due to contamination of cysteine additives and because a previous study has demonstrated no significant increase in plasma cysteine or glutathione levels when very low birth weight preterm infants received a cysteine dose of 81 mg/kg/day versus 45 mg/kg/day [25]. The most recent Cochrane review also found no benefit with regard to clinical outcomes related to cysteine supplementation [26]. Finally, a concentration of 0.5 mEq/dL (0.25 mmol/dL) of magnesium was added to all solutions. Increasing magnesium concentrations in solutions can decrease the concentrations of Ca and Phos that appear to be compatible [27–28].

A strength of the study presented in this paper is that multiple methods for particle size analyses for compounded PN solutions were performed, thus presenting more complete data over the spectrum from 1 nm to >50 microns. This may provide a more accurate depiction of the total particle size distribution for the solutions studied. The study also includes values of various measures of compatibility for control solutions containing TrophAmine without added Ca and Phos, as well as, when only Ca or only Phos were included in the PN solution. Lastly, solutions were incubated at 37°C for 24 hours prior to analysis in order to simulate conditions in a worst case scenario that might promote precipitation. Increased temperature has been shown to increase precipitation of calcium phosphates [10].

In conclusion, this study identifies concentrations of CaCl_2_ and NaPhos that appear to be compatible in neonatal PN solutions containing TrophAmine and can allow for provision of adequate intravenous intakes of calcium and phosphorus. The maximum compatible concentrations of elemental calcium, as CaCl_2_, and phosphate, as NaPhos, respectively, that could be added to AA solutions containing at least 2.5% AA were 10 mmol/L of Ca and 7.5 mmol/L of Phos for solutions without cysteine, and 10 mmol/L of Ca and 10 mmol/L of Phos for solutions containing 50 mg/dL of cysteine. This study presents data which can be used to evaluate the risk versus benefit of using calcium chloride as the calcium additive in neonatal PN solutions in North America. Other additive options that have been available in Europe that can limit the Al intake from contamination of Ca additives such as using CaGluc in plastic vials [9] or using CaCl_2_ compounded with organic phosphates that significantly increase the solubility of calcium and phosphate in PN solutions [29–31] would be preferable over current options available in North America and are very much needed.

## Supporting Information

S1 File Microflow Imaging Data-Study 1. (XLS)

S2 File Microflow Imaging Data-Study 2. (XLS)
